# The contribution of metamemory beliefs to the font size effect on judgments of learning: Is word frequency a moderating factor?

**DOI:** 10.1371/journal.pone.0257547

**Published:** 2021-09-20

**Authors:** Tian Fan, Jun Zheng, Xiao Hu, Ningxin Su, Yue Yin, Chunliang Yang, Liang Luo

**Affiliations:** 1 Collaborative Innovation Center of Assessment for Basic Education Quality, Beijing Normal University, Beijing, China; 2 Institute of Developmental Psychology, Faculty of Psychology, Beijing Normal University, Beijing, China; University of Zurich, SWITZERLAND

## Abstract

Previous studies found that metamemory beliefs dominate the font size effect on judgments of learning (JOLs). However, few studies have investigated whether beliefs about font size contribute to the font size effect in circumstances of multiple cues. The current study aims to fill this gap. Experiment 1 adopted a 2 (font size: 70 pt *vs*. 9 pt) * 2 (word frequency (WF): high *vs*. low) within-subjects design. The results showed that beliefs about font size did not mediate the font size effect on JOLs when multiple cues (font size and WF) were simultaneously provided. Experiment 2 further explored whether WF moderates the contribution of beliefs about font size to the font size effect, in which a 2 (font size: 70 pt *v*s. 9 pt, as a within-subjects factor) * 2 (WF: high *vs*. low, as a between-subjects factor) mixed design was used. The results showed that the contribution of beliefs about font size to the font size effect was present in a pure list of low-frequency words, but absent in a pure list of high-frequency words. Lastly, a meta-analysis showed evidence supporting the proposal that the contribution of beliefs about font size to the font size effect on JOLs is moderated by WF. Even though numerous studies suggested beliefs about font size play a dominant role in the font size effect on JOLs, the current study provides new evidence suggesting that such contribution is conditional. Theoretical implications are discussed.

## Introduction

Judgments of learning (JOLs) refer to people’s metacognitive predictions regarding the likelihood of remembering studied items on a later memory test, which is an important form of metamemory monitoring [1]. For decades, researchers have focused on how JOLs are constructed [2], and the documented findings suggested that JOLs are inferential in nature and are based on a variety of available cues [3]. An important cue is font size [4–7]. The font size effect on JOLs refers to a well-established phenomenon that items presented in a large font size receive higher JOLs than those presented in a small font size, although font size has little influence on recall performance, reflecting a dissociation between JOLs and memory [8].

Dozens of studies have been conducted to unravel the mechanisms underlying the font size effect on JOLs. According to the dual-basis model of metacognitive judgments [3,9], JOLs are based on both nonanalytic, experience-based cues (e.g., processing fluency) and analytic, theory-based cues (e.g., metamemory beliefs). In the former process, participants apply a nonanalytic heuristic that yields an immediate “feeling of knowing”. In the latter process, participants entail an analytic, deliberate, and conscious deduction. In terms of the font size effect on JOLs, large font size might increase JOLs through experience-based processing fluency (i.e., large items are perceived more fluently than small ones). In addition, large font size might increase JOLs through theory-based processes (i.e., metamemory beliefs that large items are easier to remember than small ones).

Recent studies suggested that theory-based processes may dominate the font size effect on JOLs [10]. For example, Mueller et al. [6] asked a group of participants to make item-by-item JOLs for words presented in either 48 pt (large) or 18 pt (small) font size. Another group of participants was instructed to make global predictions for large and small words in a belief questionnaire. The results showed that item-by-item JOLs were higher for large words than for small ones. Along the same lines, the results from the belief questionnaire showed that participants hold *a priori* belief that large words are easier to remember than small ones, suggesting that such beliefs may contribute to the font size effect on JOLs. In addition, Mueller et al. directly evaluated the contribution of fluency to the font size effect through two measures: the response times (RTs) in a lexical decision task and the self-regulated study time. Specifically, the lexical decision task measures the time it takes for a participant to decide whether the on-screen letter string is a word or non-word. Half of the words and non-words were presented in small font and the other half were presented in large font. The results showed that RTs for large and small items did not differ significantly. In another experiment, participants were given unlimited time to study large and small words, and self-regulated study time was measured. The results showed that study time did not differ significantly between large and small items. Both measures demonstrated no differences in processing fluency between large and small words, suggesting that processing fluency tends to be irresponsible for the font size effect on JOLs. Based on these findings, Mueller et al. [6] claimed that font size affects JOLs mainly through metamemory beliefs. Following studies employed different methods to further examine whether beliefs about font size contribute to the font size effect on JOLs [4,11,12].

To directly delineate the relationship between JOLs and beliefs, Hu et al. [4] conducted a participant-level regression analysis, in which the difference in JOLs between large and small words (difference in JOLs) was regressed on the difference in belief-based predictions between these categories of stimuli (difference in beliefs). The results showed that the difference in beliefs successfully predicted the difference in JOLs across participants, which supports the belief hypothesis underlying the font size effect. Several recent studies implemented multilevel linear regression models to examine whether beliefs about font size contribute to the font size effect on JOLs [11,12]. For example, Su et al. [11] applied a multilevel moderation analysis and demonstrated that the effect of font size on JOLs was significantly moderated by beliefs about font size. In summary, many studies have provided convergent evidence supporting the important role of metamemory beliefs in the font size effect.

Compatible with dual-process model, Mueller and colleagues proposed the analytic-processing theory, which provides a unique and more detailed account regarding how beliefs may influence JOLs, but does not reject the possible contribution of fluency [13,14]. According to the analytic-processing theory, when people are instructed to make judgments about future memory performance, they engage in an analytic problem-solving mode in which they attempt to reduce uncertainty by searching for cues that may be related to memory. If they can develop a plausible explanation for why a cue may influence memory or retrieve a previously developed explanation from long-term memory, then they will use beliefs about the cue when constructing JOLs. If not, then other factors will drive JOLs (most notably the difference in processing fluency that may arise as people study each item) [14].

The analytic-processing theory emphasizes the contribution of beliefs to JOL formation. In studies which manipulated only single factor such as font size [6] or pair type [14], the variability across items was easy to be detected. Take the font size effect as an example: people presumably notice that some words are presented in large size and others shown in small size, which in turn stimulates them to retrieve their pre-existing beliefs regarding how font size relates to memory performance (e.g., large words are easier to remember than small ones). People then apply such beliefs to make JOLs. However, in natural learning situations, learners frequently encounter multiple cues rather than a single cue. When dealing with multiple cues in making JOLs, people might give different weight to different cues [15]. Will participants engage in the analytic process in which they retrieve a specific belief about how one certain cue influences memory to make JOLs in circumstance of multiple cues? The current study targets to investigate this question. Below, we will briefly summarize previous evidence about whether and how multiple cues affect metacognitive judgments.

Over last years, considerable evidence regarding the impact of various cues on metacognitive judgments has accumulated. By comparison, the question about whether and how multiple cues combine to affect metacognitive judgments has received less attention [16]. Undorf and colleagues [15] are arround the first to explore whether multiple cues jointly affect JOLs. They systematically investigated whether participants integrate multiple extrinsic and intrinsic cues in JOLs. They varied two extrinsic cues (font size and number of study presentations) in Experiment 1, and found that participants integrated both cues in their JOLs. In Experiment 2, they demonstrated that participants could integrate two intrinsic cues (concreteness and emotionality) in their JOLs. When manipulating all four factors simultaneously in Experiment 3, Undorf and colleagues observed that participants could integrated all four cues in their JOLs. Finally, Experiment 4 manipulated font size, concreteness, and emotionality in a continuum rather than in two easily distinguishable levels, and the results showed successful integration of these three cues in JOLs. In conclusion, Undorf and colleagues provided important findings suggesting that participants have a remarkable capacity to integrate multiple cues to construct JOLs.

Later, Undorf and Bröder demonstrated that cue integration was more likely due to strategic integration of multiple cues, rather than reliance on a single unified feeling of ease [17]. In their study, concreteness and emotionality simultaneously acted on both pre-study JOLs (i.e., JOLs that are made before encoding each item, generally reflecting one’s metamemory beliefs) and immediate item-by-item JOLs (i.e., JOLs that are made immediately following encoding each item). These findings imply that metamemory beliefs may contribute to JOL formation in situations of multiple cues (see [17], p. 640).

Another important study of Price and Harrison simultaneously manipulated multiple cues (font size & item relatedness) and explored the bases of JOLs [18]. They collected pre-study JOLs, immediate JOLs, and the combination of both types of JOLs to explore the bases of JOL formation (beliefs or fluency). Their results revealed that, compared to pre-study JOLs, immediate JOLs demonstrated a larger effect of relatedness and a smaller effect of font size. In addition, immediate JOLs showed greater alignment with recall performance, as reflected by higher relative accuracy for immediate JOLs than that for pre-study JOLs in all three experiments. As pre-study JOLs are mainly based on metamemory beliefs [2,6], the difference between pre-study JOLs and immediate JOLs implies that immediate JOLs are likely based on other factors, besides metamemory beliefs.

The above-discussed studies [17,18] tend to suggest that beliefs may contribute to JOL formation in circumstances of multiple cues. However, these studies suffer from limitations in research methods. Based on the comparison between pre-study JOLs and immediate JOLs, researchers can only indirectly conjecture whether or not beliefs contribute to JOLs in circumstances of multiple cues. There is no direct evidence regarding whether beliefs actually contribute to JOL formation in such situations. In other words, concurrently observing that pre-study and standard JOLs vary in the same direction between different levels of a given factor (e.g., large and small font size) is insufficient to conclude that beliefs contribute to JOL construction. Recent studies advocate directly testing whether beliefs (i.e., pre-study JOLs) statistically mediate that factor’s effect on standard JOLs [2,19]. Hence, the current study aims to employ mediation analysis to measure a certain belief’s contribution to JOL formation under circumstances of multiple cues.

The current study focused on the contribution of *a priori* beliefs about font size to the font size effect on JOLs. Apart from font size, the current study chose word frequency (WF) as another manipulated factor. WF is an inherent characteristic of words and is easy to be manipulated. Moreover, WF exerts robust effects on memory and metamemory judgments. For instance, WF strongly relates to the feeling of familiarity [20]. High-frequency words are perceived as more familiar and are easier to be retained in working memory [21–23]. Previous studies also demonstrated that JOLs are sensitive to WF. Fiacconi and Dollois conducted a meta-analysis to investigate the effect of WF on JOLs [24]. They found a reliable effect of WF on JOLs through integrating results across 17 experiments, with high-frequency words receiving higher JOLs than low-frequency ones. As WF is a reliable factor affecting both memory and metamemory judgments, it is reasonable to manipulate it as another factor in the current study.

As both the dual-process model and the analytic-processing theory assume that beliefs about font size contribute importantly to the font size effect when font size is the only manipulated factor [4,6,11], the current study hypothesized that beliefs about font size may contribute to the font size effect even in circumstance of multiple cues. However, it is also reasonable to expect a minimal role of beliefs about font size in the font size effect. For instance, when dealing with multiple cues (e.g., font size and WF) to make JOLs, participants might assign higher weight to WF than to font size, perhaps because WF is considered more diagnostic of future recall performance than font size. Participants might then pay less attention to font size and consequently might not take pains to retrieve a previously developed explanation about how font size influences memory performance. Therefore, it is possible that beliefs about font size do not mediate the font size effect on JOLs in circumstance of multiple cues.

These two alternative hypotheses were tested in Experiment 1, in which a 2 (font size: large *vs*. small) * 2 (WF: high *vs*. low) within-subjects design was employed. WF was manipulated within-subjects in order to serve as the additional cue for JOLs. In this circumstance of multiple cues, whether participants’ pre-existing beliefs about font size contribute to the font size effect was investigated. In Experiment 2, WF was changed as a between-subjects variable, which would no longer serve as an apparent cue for JOLs. Specifically, one group of participants studied a pure list of high-frequency words, and another group of participants studied a pure list of low-frequency words. According to the analytic-processing theory, with font size as the single within-subjects manipulated factor, participants could easily detect font size as an available cue for JOLs and their pre-existing beliefs about font size would be activated, which in turn drives JOLs. If this were true, beliefs about font size would contribute to the font size effect on JOLs in both high-frequency and low-frequency conditions. However, one may doubt if results would differ in high-frequency and low-frequency conditions, as high-frequency and low-frequency words naturally differ in ease of encoding and association to one another [22,23]. In this way, the current study further asks if WF per se moderates the contribution of beliefs about font size to the font size effect on JOLs.

## Experiment 1

The purpose of Experiment 1 was to investigate whether beliefs about font size contribute to the font size effect on JOLs in circumstance of multiple cues. In Experiment 1, the procedure was adapted from that of Hu et al.’s Experiment 2 [4]. On the first day, participants made belief-based predictions regarding the relationship between font size and memory in a questionnaire. Twenty-four hours later, they studied large (70 pt) and small (9 pt) words and provided item-by-item JOLs. Participants were not informed about the manipulation of WF.

### Method

#### Participants

A power analysis was conducted using G*Power to determine the required sample size [25]. According to the effect sizes of the font-size effect documented in previous studies (*η*_p_^2^ ranging from 0.13 to 0.50 [7]), 6–24 participants are required to obtain a significant (*α* = 0.05) font-size effect at 95% power. Statisticians suggest that when multilevel analysis is performed, the data should be collected from at least 30 participants with more than 30 trials for each participant [26]. In this way, 32 students (25 women; mean age = 21.19, *SD* = 1.89) were recruited from Beijing Normal University (BNU). Each participant was tested individually in a sound-proofed cubicle, provided written consent, and received 25 RMB as compensation. Experiments 1 and 2 were approved by the Ethics Committee at the BNU Collaborative Innovation Center of Assessment for Basic Education Quality.

#### Design & materials

A 2 (font size: large *vs*. small) * 2 (WF: high *vs*. low) within-subjects design was used. The principal stimuli consisted of 42 two-character concrete Chinese words extracted from the Chinese word database developed by Cai and Brysbaert [27]. Two words were used for practice and were excluded from data analyses. The remaining 40 words were used in the formal experiment, of which 20 were high-frequency words (with WF ranging from 35.29 to 363.23 per million) and the other 20 were low-frequency words (with WF ranging from 0.06 to 1.28 per million). High-frequency (mean WF = 130.14, *SD* = 101.28) and low-frequency (mean WF = 0.67, *SD* = 0.38) words differed significantly in WF, *t*(19.00) = 5.72, *p* < .001, Cohen’s *d* = 1.81.

High-frequency words were randomly divided into two sets, with 10 words in each set. One set was presented in 9 pt (small size), with the other set presented in 70 pt (large size). Set assignment to the font size conditions was counterbalanced across participants. The two sets of high-frequency words did not differ significantly in WF or number of strokes (*p*s > .20). Low-frequency words were also randomly divided into two sets, with one set presented in large font and the other in small font, and set assignment was counterbalanced across participants. The two sets of low-frequency words did not differ significantly in WF or number of strokes (*p*s > .80). In summary, for each participant, there were 10 high-frequency words in 70 pt, 10 high-frequency words in 9 pt, 10 low-frequency words in 70 pt, and 10 low-frequency words in 9 pt.

#### Procedure

The experiment took place over two consecutive days and consisted of two tasks: (a) a belief judgment task and (b) a study-test task. On the first day, participants undertook a belief judgment task similar to that in Hu et al.’s Experiment 2 [4]. They read the descriptions of a study-test task and saw the rectangles that represented 70 pt and 9 pt font. They were instructed to imagine that they were attending that task and were asked to estimate the numbers of large and small words (out of 20) that they would be able to remember on a later memory test. The order of estimates for large and small words was counterbalanced across participants. This belief questionnaire is delivered one day before the learning task in order to get a pure measure of participants’ *a priori* beliefs about how font size influences memory performance.

Twenty-four hours later, participants returned to the same laboratory room and took the study-test task. During the study phase, participants studied high-frequency and low-frequency words one-by-one, and these words were presented in either 70 pt or 9 pt. The 40 words were presented in a pseudorandom order with no more than 3 words in the same font size or WF presented consecutively. Each trial began with a blank white screen presented for 500 ms, followed by a word presented at the center of a white screen for 4000 ms. Immediately following the presentation of each word, participants were asked to make a JOL on a scale ranging from 0 (*Sure I will not remember it*) to 100 (*Sure I will remember it*). They typed their JOLs into the computer. There was no time limitation for participants to make JOLs. After typing their JOLs, participants pressed ENTER and the next trial initiated.

**Fig 1:**
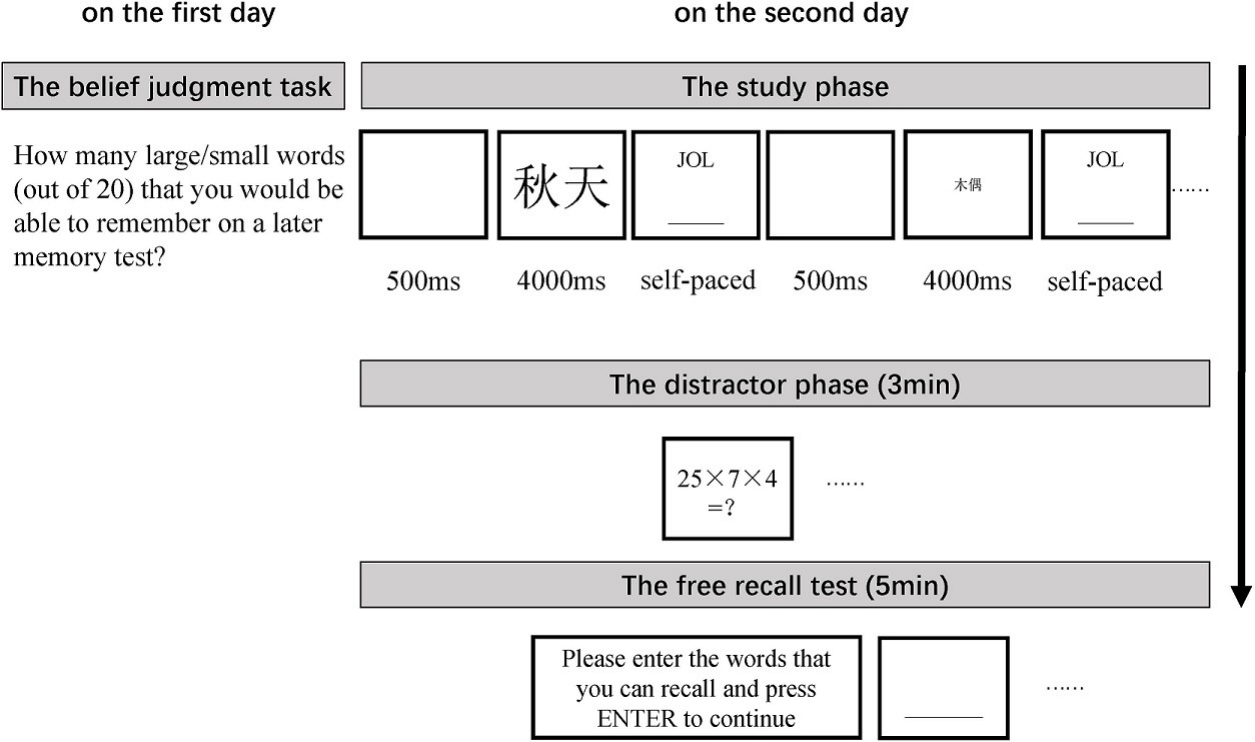
Experimental procedure for Experiment 1.

Following the study phase, a distractor phase was initiated, during which participants were instructed to solve as many arithmetic problems as they could in 3 min. Finally, participants undertook a free recall test, wherein they recalled as many words as they could in 5 min and typed their answers into the computer. No feedback was offered during the test. The experimental procedure is shown in Fig 1.

### Results and discussion

#### Effects of font size (and WF) on belief-based predictions, JOLs, and recall performance

Table 1 summarizes *Mean*s and *SD*s of JOLs and test performance as functions of font size and WF. Predictions in the belief judgment task and test performance were transformed into percentages. For JOLs, there were several erroneous inputs (e.g., greater than 100) in the current and following experiments, which were treated as missing data. In Experiments 1 and 2, the missing rates (< 0.54%) of JOL data were minimal.

**Table 1:** *Means* (*SDs*) for JOLs and recall performance in Experiment 1.

WF	high-frequency		low-frequency	
Font size	large	small	large	small
JOL (%)	68.11 (15.05)	62.87 (16.24)	45.18 (19.23)	39.19 (18.13)
Recall (%)	57.81 (19.30)	52.19 (18.27)	31.25 (16.61)	32.19 (16.21)

**Note.** JOL = judgment of learning; WF = word frequency.

In the questionnaire, participants predicted that they would remember more large words (*M* = 53.28, *SD* = 13.83) than small ones (*M* = 38.75, *SD* = 13.68), *t*(31) = 10.89, *p* < .001, Cohen’s *d* = 1.92. In the study-test task, a repeated-measures analysis of variance (ANOVA), with font size (large *vs*. small) and WF (high *vs*. low) as the within-subjects independent variables and JOLs as the dependent variable, showed that participants gave higher JOLs to large words (*M* = 56.63, *SD* = 15.91) than to small ones (*M* = 51.08, *SD* = 15.73), *F*(1,31) = 12.92, *p* = .001, *η*_p_^2^ = 0.29. In addition, JOLs were higher for high-frequency words (*M* = 65.47, *SD* = 14.68) than for low-frequency words (*M* = 42.18, *SD* = 17.89), *F*(1,31) = 116.22, *p* < .001, *η*_p_^2^ = 0.79. There was no significant interaction between font size and WF, *F*(1,31) = 0.12, *p* = .74, *η*_p_^2^ = 0.004.

For recall performance, a 2 (font size: large *vs*. small) * 2 (WF: high *vs*. low) repeated-measures ANOVA was conducted. The results showed that participants recalled more high-frequency words (*M* = 55.00, *SD* = 15.66) than low-frequency ones (*M* = 31.72, *SD* = 12.61), *F*(1,31) = 130.17, *p* < .001, *η*_p_^2^ = 0.81. Recall performance did not differ between large (*M* = 44.53, *SD* = 16.13) and small words (*M* = 42.19, *SD* = 14.08), *F*(1,31) = 0.73, *p* = .40, *η*_p_^2^ = 0.02. There was no significant interaction between font size and WF, *F*(1,31) = 1.77, *p* = .19, *η*_p_^2^ = 0.05.

#### Individual-level analysis of the effects of font size and WF on JOLs

An individual-level analysis focusing on cue utilization was conducted [15]. Participants were coded as reliably basing JOLs on font size if their JOLs were higher for large words than for small ones. Likewise, participants were coded as reliably basing their JOLs on WF if their JOLs were higher for high-frequency words than for low-frequency ones. Meanwhile, effect sizes were also taken into consideration for reliable cue effects in these expected directions and Cohen’s *d* ≥ 0.20 for small effects is used as a criterion [28].

Sixteen participants (50%) revealed *d*s ≥ 0.20 for both cues, which is indicative of reliably basing JOLs on both font size and WF. Fourteen participants (43.75%) mainly focused on WF, as indicated by *d* ≥ 0.20 for the WF effect and *d* < 0.20 for the font size effect. One participant (3.13%) mainly focused on font size, as indicated by *d* ≥ 0.20 for the font size effect and *d* < 0.20 for the WF effect. One participant (3.13%) revealed *d*s < 0.20 for both cues.

#### Contribution of beliefs about font size to the font size effect on JOLs

The main research interest of the current study was to explore whether beliefs about font size contribute to the font size effect on JOLs in circumstance of multiple cues. To explore this question, a multilevel mediation analysis was conducted to explore whether and to what extent the font size effect on JOLs was mediated by beliefs about font size. In addition, Bayes Factor (BF) was computed using the statistical software program JASP [29] to estimate the strength of the evidence for non-significant effects.

Although the belief-based predictions remained the same within the same level of font size, there were still variations for beliefs across cue levels for each participant. Thus, *Belief* was treated as a variable at the item level in the current multilevel models [19]. In addition, WF was added as an item-level moderator and a moderated mediation analysis was conducted.

**Fig 2:**
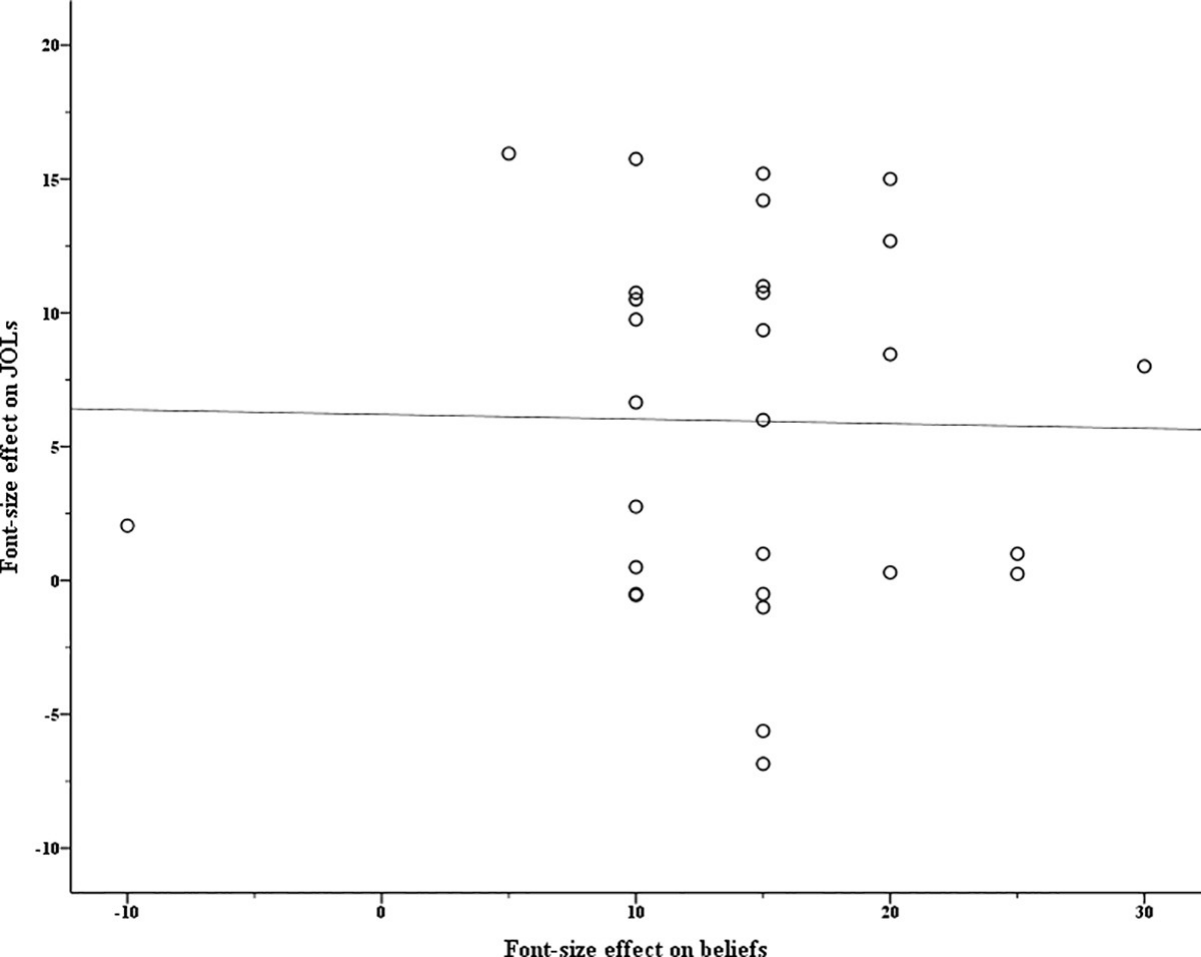
The relationship between font-size effects on beliefs and JOLs in Experiment 1. The font-size effect on beliefs is measured as the difference in belief-based predictions between large and small words. The font-size effect on JOLs is calculated as the difference in mean JOLs between large and small words. Each point represents one individual participant.

As a single observation (e.g., an outlier) can have a substantial influence on the results of a regression analysis [30], it is important to detect influential observations before conducting the regression analysis. In the current research, difference in fits (DFFITS) were used to identify influential data points. DFFITS measures how much an observation affects its fitted value from the regression model. It is a widely-used quantitative measure to detect outliers, better than scatterplots in assessing data points [31]. The R *olsrr* package was used to depict DFFITS to figure out the influential observations (see analysis code online: *detect outliers*.*R*). In Experiment 1, three participants were detected as outliers and were hence excluded from further multilevel mediation analysis. For the remaining 29 participants, Fig 2 shows the relationship between font-size effects on beliefs and JOLs [4].

Experiment 1 used the R *lme4*, *lmerTest*, and *mediation* packages to conduct multilevel regression and mediation analyses (Level 1: items; Level 2: participants) [32–34]. Missing data were excluded listwise from multilevel mediation analyses in the current and following experiments. The font size of 70 pt was coded as 1 and 9 pt was coded as 0. Meanwhile, high-frequency words were coded as 1, and low-frequency words were coded as 0. *Font Size* and *Belief* were group-mean-centered. *Belief* was regressed on *Font Size* in the first model. The random slope of *Font Size* was excluded because adding this random slope made the model fail to converge [19,35]. In the second model, *JOL* was regressed on *Font Size*, *Belief*, *WF*, and the interaction between *WF* and *Belief*. A random intercept together with the random slopes of *Font Size* and *Belief* were included. The coefficients are shown in Table 2, wherein *a* represents the effect of *Font Size* on *Belief*; *b* represents the effect of *Belief* on *JOL*; *c’* represents the direct effect of *Font Size* on *JOL* when other variables were controlled.

**Table 2:** Results from the multilevel mediation models in Experiments 1 and 2.

Effect	Estimate (*β*)	*SE*	*df*	*t* or *Z* value	*p*-value	95% CI
**Experiment 1 (*N* = 29)**						
*a*	14.14	0.21	1154.00	67.83	< .001	[13.74, 14.55]
*b*	0.04	0.21	13.63	0.18	0.86	[-0.40, 0.48]
*c’*	5.73	3.15	7.09	1.82	0.11	[-0.71, 12.47]
*WF*	22.99	1.20	1101.48	19.08	< .001	[20.62, 25.35]
WF*Belief	-0.04	0.15	1101.41	-0.25	0.81	[-0.34, 0.26]
*INDbelief*	0.51				0.86	[-5.36, 6.39]
**Experiment 2 (high-frequency group; *N* = 47)**						
*a*	13.40	0.23	1875.00	58.13	< .001	[12.95, 13.86]
*b*	0.03	0.10	51.75	0.28	0.78	[-0.17, 0.22]
*c’*	3.62	1.58	472.08	2.29	0.02	[0.51, 6.72]
*INDbelief*	0.36	1.32		0.28	0.78	[-2.28, 2.91]
**Experiment 2 (low-frequency group; *N* = 50)**						
*a*	16.09	0.27	1986.00	59.93	< .001	[15.57, 16.62]
*b*	0.19	0.09	49.82	2.08	0.04	[0.01, 0.38]
*c’*	4.78	1.84	49.70	2.60	0.01	[1.09, 8.48]
*INDbelief*	3.07	1.48		2.08	0.04	[0.18, 6.02]
**Experiments 2 (*N***_**high-frequency**_ **= 47, *N***_**low-frequency**_ **= 50, with WF as a participant-level moderator)**						
*a*	14.79	0.18	3863.00	82.57	< .001	[14.44, 15.14]
*b*	0.22	0.07	40.86	2.91	0.006	[0.07, 0.37]
*c’*	4.18	1.15	319.64	3.64	< .001	[1.92, 6.44]
*WF*	4.27	3.29	95.01	1.30	0.20	[-2.26, 10.79]
WF*Belief	-0.22	0.08	42.49	-2.66	0.01	[-0.39, -0.05]
*INDbelief*	3.20	1.10		2.91	0.004	[1.03, 5.39]

**Note.***SE*, standard error; *df*, degree of freedom; CI, confidence interval; *WF*, word frequency; *INDbelief*, the indirect effect of *Font Size* on *JOL* through *Belief*. For *INDbelief*, *Z* value was reported.

The R *mediation* package was used to conduct mediation analyses [34]. As shown in Table 2, the indirect effect of font size on JOLs through beliefs about font size was not statistically significant when WF was controlled, and the interaction between WF and beliefs was non-significant either, implying that WF did not moderate the contribution of beliefs about font size to the font size effect on JOLs in this experiment (see data and analysis code online).

In order to explore the reliability of the non-significant result about the contribution of beliefs about font size to the font size effect, the Bayesian linear regression was conducted. The difference in belief-based predictions between large and small words (difference in beliefs) was used as the predictor variable. The difference in mean JOLs between large and small words (difference in JOLs) was used as the outcome variable. All the default prior settings in JASP was used [29]. BF_10_ is a measure of the fit of the data under the alternative model relative to the fit under the null model. Larger BF_10_ values reflect more support for the alternative model versus the null model. Its inverse, BF_01_ = 1/BF_10_, indicates the strength of the evidence for the null model versus the alternative model. In Experiment 1, the data are 2.86 times more likely under the null model compared to a regression model including difference in beliefs (BF_01_ = 2.86).

In summary, when WF was manipulated within-subjects in order to serve as an additional cue, the results revealed that participants’ beliefs about font size did not mediate the font size effect on JOLs. This result was inconsistent with findings from previous studies [4,11]. We re-analysed data from two previous studies: Hu et al.’s Experiment 2 and Su et al.’s Experiment 2b. Similar to our study, they measured participants’ pre-existing beliefs about font size on the first day. On the second day, participants took a study-test task in which JOLs were measured. Su et al.’s study focused on the simultaneous contributions of ease of learning judgments (EORs) and pre-existing beliefs about font size on JOLs in their Experiments 2a and 2b. In Su et al.’s Experiment 2b, EORs were given after participants had studied and made JOLs, which eliminated the possible influence of EORs on JOLs. Meanwhile, the study time was fixed to 5s per item rather than self-paced. As Su et al.’s Experiment 2b was more similar with our study, we only re-analysed their Experiment 2b data.

Our re-analyses of both studies showed that the indirect effect of font size on JOLs through beliefs about font size was statistically significant (as shown in S1 Table). As the experimental procedure in the current study is similar to those used in previous two studies, we look in details about possible difference in study materials. The median WF in Hu et al.’s study was 20.24 per million words, and was 2.97 per million words in Su et al.’s study. In our Experiment 1, the median WF was 89.00 per million words for high-frequency words and was 0.71 per million words for low-frequency words. Compared to high-frequency words used in the current study, study materials used in both Hu et al.’s and Su et al.’s studies could be categorized as low-frequency words. More importantly, WF was controlled in a small range in their studies and WFs were highly similar for words presented in small or large sizes. In a word, both Hu et al.’s and Su et al.’s studies support the idea that beliefs about font size contribute to the font size effect in a relatively pure list of low-frequency words. However, in the current experiment, WF was manipulated within-subjects. In this circumstance of multiple cues (font size & WF), participants’ pre-existing beliefs about font size contributed minimally to the font size effect on JOLs.

As far as we know, the current study should be the first to provide evidence suggesting that people’s pre-existing beliefs about font size contributed little to the font size effect in circumstance of multiple cues. Compared with results from previous studies, it is possible that the manipulation of WF provided participants with another robust cue for inferencing JOLs, which could have been used as the more dominant cue for JOLs. Participants might then pay less attention to font size and consequently might not take pains to retrieve their beliefs about font size to make JOLs.

In Experiment 2, WF was changed as a between-subjects variable, which would no longer serve as an apparent cue to influence JOLs. Specifically, one group of participants studied a pure list of high-frequency words, and another group of participants studied a pure list of low-frequency words. In each group, WF was controlled in a relatively small range. With font size as a single within-subjects factor, Experiment 2 aimed to figure out if beliefs about font size contribute to the font size effect in both high-frequency and low-frequency conditions. As high-frequency words and low-frequency words naturally differ in semantic characteristics [22,23], Experiment 2 further investigated whether WF per se is a moderating factor of the contribution of beliefs about font size to the font size effect.

## Experiment 2

In Experiment 2, the key question was whether WF moderates the contribution of beliefs about font size to the font size effect when it is manipulated between-subjects.

### Method

#### Participants

Statisticians suggest that when we perform multilevel analysis, we should collect data from at least 30 participants, and it would be better to collect data from 50 participants [26]. As moderated mediation analysis would be conducted in Experiment 2, at least 50 participants in each group are expected. In this way, 105 participants were recruited from BNU. Fifty-three students studied a pure list of high-frequency words (high-frequency group), with 36 women and mean age = 21.08 (*SD* = 2.46). The other 52 students studied a pure list of low-frequency words (low-frequency group), with 39 women and mean age = 20.88 (*SD* = 2.65). Each participant was tested individually and received 30 RMB as compensation. All participants provided written consent.

#### Design and materials

Experiment 2 involved a 2 (font size: large *vs*. small) * 2 (WF: high *vs*. low) mixed design, with font size as a within-subjects variable and WF as a between-subjects variable. The stimuli in the high-frequency group consisted of 42 two-character high-frequency Chinese words selected from the Chinese word database developed by Cai and Brysbaert [27]. Two of them were used for practice and were excluded from data analyses. WF of the remaining 40 concrete words ranged from 78.58 to 214.87 per million (*Mean* = 127.35, *SD* = 37.43). WF did not differ significantly between high-frequency words in Experiment 2 and those in Experiment 1, *t*(21.64) = 0.12, *p* = .91, Cohen’s *d* = 0.04.

The stimuli in the low-frequency group consisted of 42 two-character low-frequency Chinese words chosen from the same word database [27]. Two words were used for practice and were excluded from data analyses. WF of the remaining 40 concrete words ranged from 0.39 to 1.07 per million. The average of WF in the low-frequency group was 0.67 (*SD* = 0.22), which did not differ significantly from that for low-frequency words in Experiment 1, *t*(25.61) = 0.06, *p* = .95, Cohen’s *d* = 0.02.

Words in both the high-frequency group and low-frequency groups were randomly divided into two sets, with 20 words in each set. The two sets did not differ significantly in WF or number of strokes (*p*s > .30). One set was presented in 9 pt, and the other set was presented in 70 pt. Set assignment was counterbalanced across participants.

#### Procedure

The procedure in Experiment 2 was the same as that in Experiment 1, except that participants studied a pure list of high-frequency or low-frequency words.

### Results and discussion

#### Effects of font size (and WF) on belief-based predictions, JOLs, and recall performance

The *Mean*s and *SD*s of belief-based predictions, JOLs, and recall performance for large and small words are summarized in Table 3.

**Table 3:** *Means* (*SDs*) for belief-based predictions, JOLs, and recall performance in Experiment 2.

	font size	
	large	small
**Experiment 2 (high-frequency group)**		
Belief (%)	55.19 (14.34)	40.00 (15.29)
JOL (%)	54.34 (15.90)	48.86 (18.03)
Recall (%)	52.36 (17.25)	50.66 (15.96)
**Experiment 2 (low-frequency group)**		
Belief (%)	55.38 (15.33)	39.33 (15.18)
JOL (%)	52.24 (16.70)	44.31 (15.73)
Recall (%)	36.06 (18.51)	34.04 (20.39)

**Note.** JOL = judgment of learning.

In the questionnaire, a 2 (font size: large vs. small, within-subjects variable) * 2 (group: high-frequency group vs. low-frequency group, between-subjects variable) mixed ANOVA was conducted, with belief-based predictions as the dependent variable. Participants estimated that they would remember more large words than small ones, *F*(1, 103) = 172.35, *p* < .001, *η*_p_^2^ = 0.63. There was no significant difference in the belief-based predictions between high-frequency and low-frequency groups, *F*(1, 103) = 0.008, *p* = .93, *η*_p_^2^ < 0.001. There was no significant interaction between font size and WF, *F* (1, 103) = 0.13, *p* = .72, *η*_p_^2^ = 0.001.

In the study-test task, a 2 (font size: large *vs*. small, within-subjects variable) * 2 (WF: high *vs*. low, between-subjects variable) mixed ANOVA was conducted, with JOLs as the dependent variable. The results showed that participants provided higher JOLs to large words than to small ones, *F*(1, 103) = 68.83, *p* < .001, *η*_p_^2^ = 0.40. JOLs for high-frequency words were not significantly higher than JOLs for low-frequency words, *F*(1, 103) = 1.12, *p* = .29, *η*_p_^2^ = 0.01. This result is easy to understand because WF was manipulated between-subjects. There was no significant interaction between font size and WF, *F* (1, 103) = 2.29, *p* = .13, *η*_p_^2^ = 0.02.

For recall performance, a 2 (large *vs*. small) * 2 (high-frequency *vs*. low-frequency) mixed ANOVA was conducted. The results showed that recall performance for high-frequency words was significantly higher than that for low-frequency words, *F*(1,103) = 25.20, *p* < .001, *η*_p_^2^ = 0.20. Recall performance did not differ between large words and small ones, *F*(1,103) = 2.02, *p* = .16, *η*_p_^2^ = 0.02. There was no significant interaction between font size and WF, *F*(1,103) = 0.02, *p* = .90, *η*_p_^2^ < 0.001.

#### Individual-level analysis of the font size effect on JOLs

An individual-level analysis that focused on the font size effect was conducted. Similar to that in Experiment 1, participants were coding as reliably basing JOLs on font size if their JOLs were higher for large words than for small ones and Cohen’s *d* for the font size effect on JOLs was greater than 0.20. Thirty participants (56.60%) in the high-frequency group and 34 participants (65.38%) in the low-frequency group focused on font size reliably when making JOLs. The proportion of participants who reliably focused on font size did not differ significantly between groups, *χ*^2^(1) = 0.85, *p* = .36.

#### The contribution of beliefs about font size to the font size effect on JOLs

**Fig 3:**
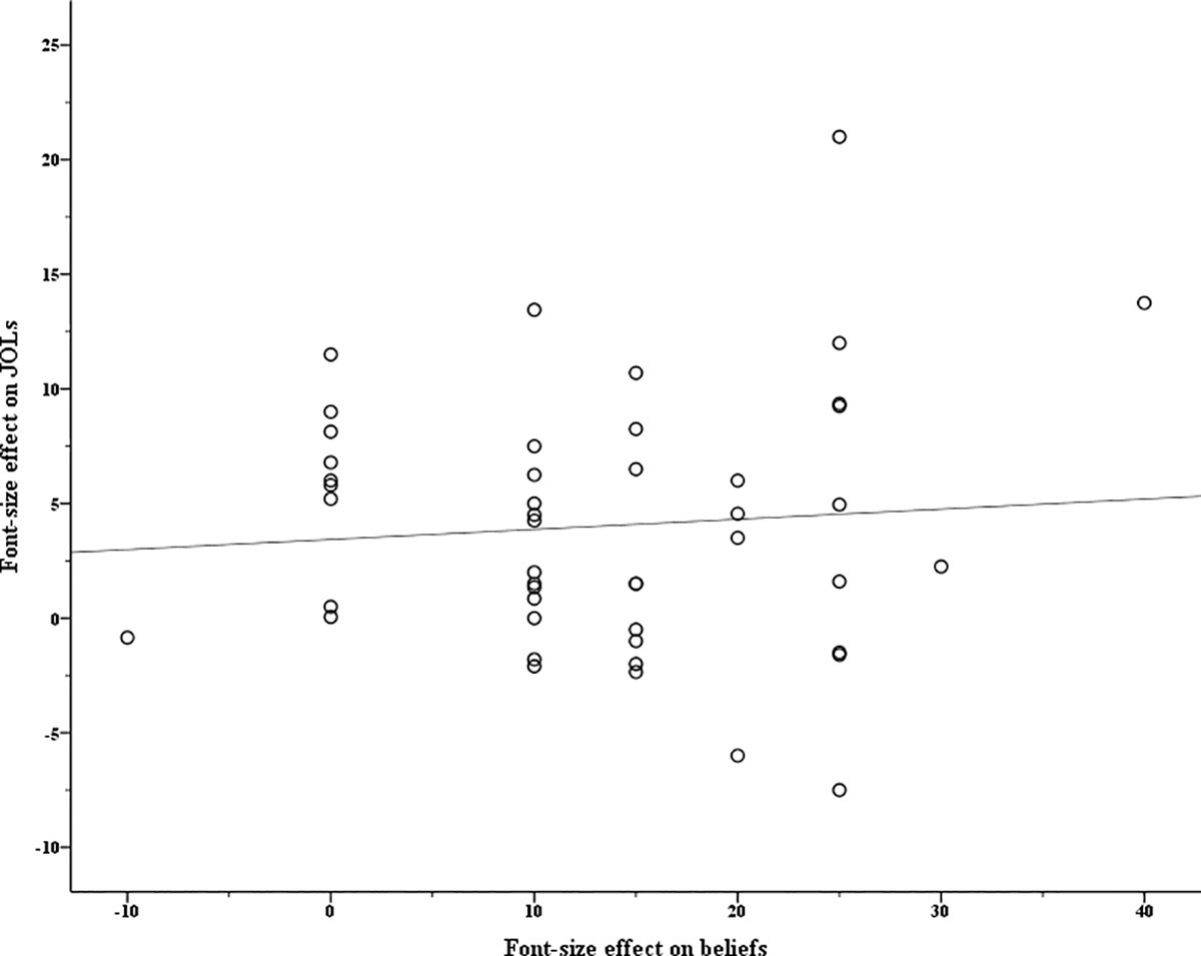
The relationship between the font size effects on beliefs and JOLs in Experiment 2’s high-frequency group.

**Fig 4:**
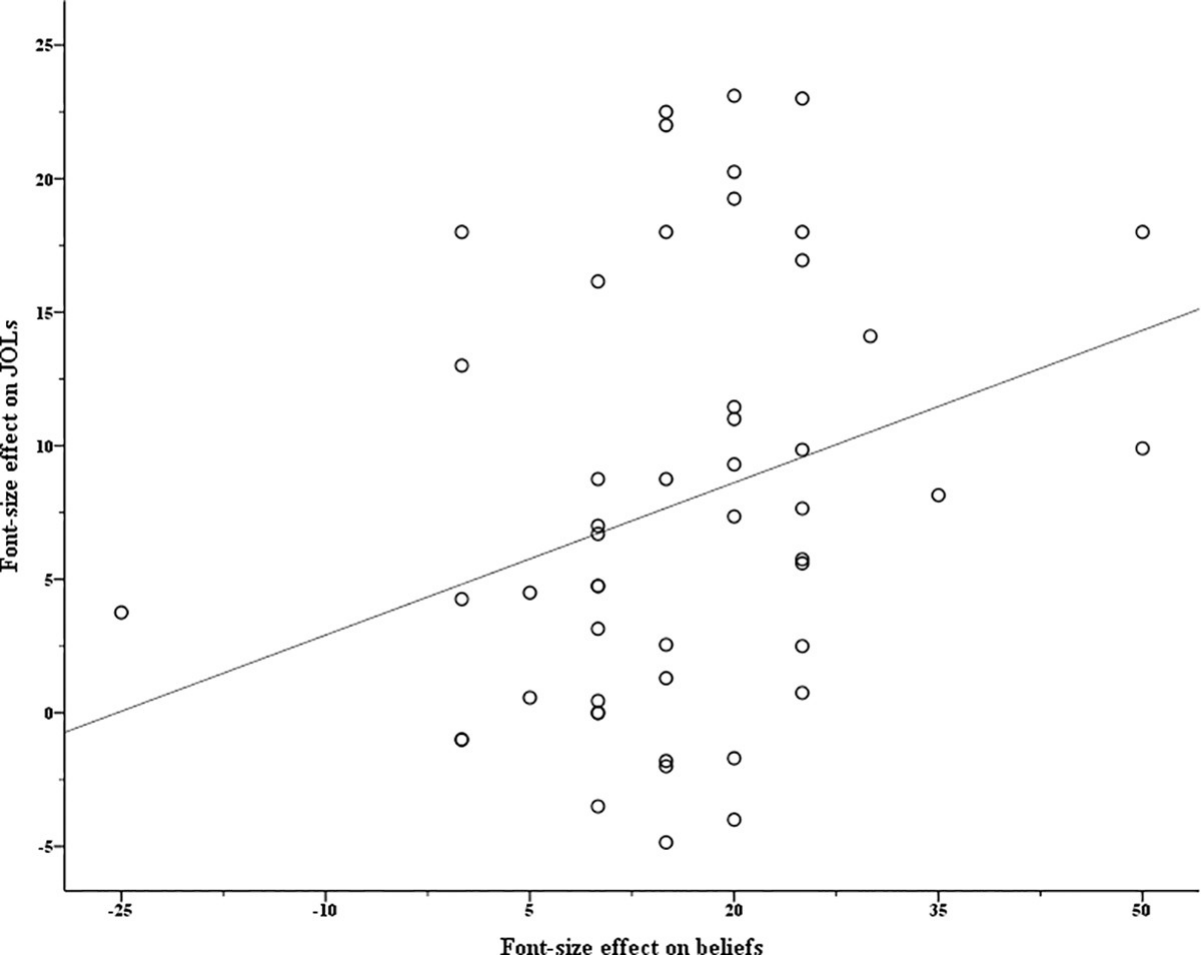
The relationship between the font size effects on beliefs and JOLs in Experiment 2’s low-frequency group.

The same procedure as that in Experiment 1 was conducted to detect influential observations (see analysis code online: *detect outliers*.*R*) for the high-frequency and the low-frequency groups, respectively. Six participants in the high-frequency group and two participants in the low-frequency group were detected as outliers and were excluded from further regression analyses. For the remaining 47 participants in the high-frequency group and 50 participants in the low-frequency group, Figs 3 and 4 show the relationship between the font size effects on beliefs and JOLs, respectively [4]. Later, the multilevel mediation analyses were performed with the MLmed macro in SPSS (Level 1: items; Level 2: participants; [36]) for the high-frequency group and the low-frequency group, respectively.

The font size of 70 pt was coded as 1 and 9 pt was coded as 0. *Font size* and *Belief* were group-mean-centered. When regressing *Belief* on *Font Size*, the random intercept was removed because *Belief* was group-mean-centered, and the averages of beliefs across all trials should always be zero for each participant. The random slope of *Font Size* on *Belief* was also removed, because the belief-based predictions were the same for all trials within a certain level of font size for a participant, and adding this random slope made the model fail to converge. When *JOL* were regressed on *Font Size* and *Belief*, the random intercept and random slopes for the effect of *Font Size* and *Belief* on *JOL* were included into the model, except when a random slope could not be estimated by SPSS (which suggests that there might be no random effect for the slope) [37].

The results from the multilevel mediation model are shown in Table 2. In the high-frequency group, the indirect effect of font size on JOLs through beliefs about font size was non-significant. In the low-frequency group, the effect of font size on JOLs was significantly mediated by beliefs about font size.

To explore the reliability for the non-significant effect in the high-frequency group, the same Bayesian linear regression was conducted as in Experiment 1. The data are 3.06 times more likely under the null model compared to a regression model including difference in beliefs (BF_01_ = 3.06).

Later, data from the two groups in Experiment 2 were combined and a moderated mediation analysis was performed, with WF as a participant-level moderator. The results are shown in Table 2. The indirect effect of font size on JOLs through beliefs about font size was significant when WF was controlled. The interaction between *Belief* and *WF* on JOLs was also significant, implying that WF significantly moderated the contribution of beliefs about font size to the font size effect on JOLs.

In conclusion, with font size as the only within-subjects manipulated factor, Experiment 2 provided results suggesting that the font size effect on JOLs was significantly mediated by beliefs about font size in a pure list of low-frequency words, consistent with previous findings [4,11]. But this mediating effect was absent in a pure list of high-frequency words. These findings were reconfirmed by the results from the moderated mediation model, in which WF significantly moderated the contribution of beliefs about font size to the font size effect on JOLs. Below, we conducted a small scale meta-analysis to investigate the general effect of the contribution of beliefs about font size to the font size effect, and to assess whether WF serves as a moderator of the contribution of beliefs about font size.

## Meta-analysis

In the current study, the contribution of beliefs about font size to the font size effect was only present in Experiment 2’s low-frequency group, but was absent in Experiment 1 and Experiment 2’s high-frequency group. An unpublished experiment from our lab used the same low-frequency words as those in Experiment 2’s low-frequency group. The only difference between this unpublished experiment and Experiment 2’s low-frequency group was that, before the study-test task, participants performed a lexical decision task. This experiment replicated the findings in Experiment 2’s low-frequency group (See S1 Appendix for details). Meanwhile, our re-analyses of Hu et al.’s Experiment 2 and Su et al.’s Experiment 2b show that the contribution of beliefs about font size to the font size effect was significant in their studies. Since all of these studies focused on the contribution of one’s pre-existing beliefs about font size to the font size effect, it is reasonable to conduct a small-scale meta-analysis to explore the general effect. Moreover, the above studies varied in WF. Therefore, it is important to examine whether WF moderates the contribution of beliefs about font size to the font size effect.

**Fig 5:**
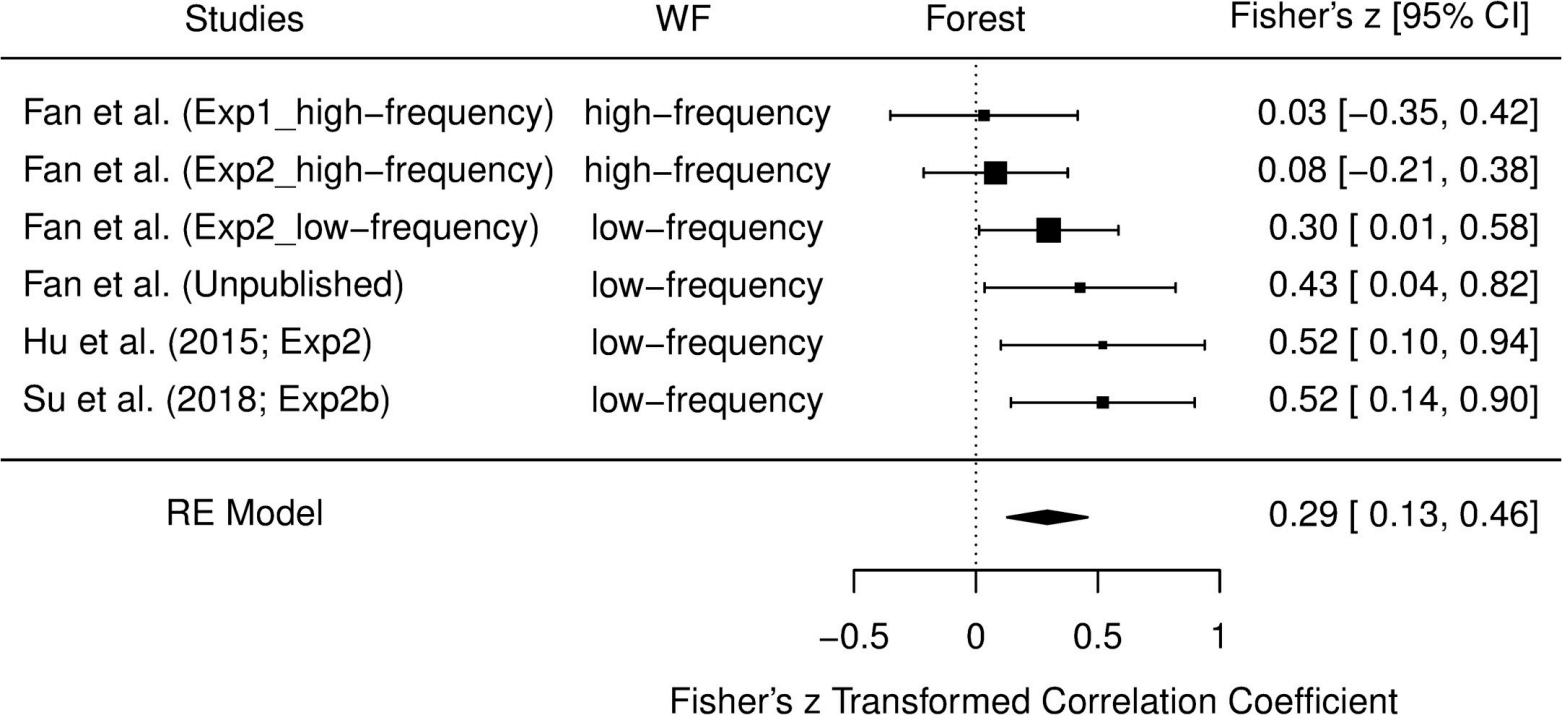
Forest plot depicting the meta-analytic summary of the correlation between beliefs about font size and the font size effect on JOLs. Each raw depicts a single experiment with its WF condition. The estimated effect sizes along with their 95% confidence intervals (CIs) are also shown. The black diamond at the bottom of the plot represents the overall effect size (Fisher’s *z*), estimated using a random-effects model. The horizontal end-points of the diamond correspond to the upper and lower limits of 95% CI.

The meta-analysis was conducted on a set of six studies as shown in Fig 5. For our Experiment 1, the data was split into high-frequency and low-frequency words separately. Only the data for high-frequency words were included in the meta-analyses for two reasons. Firstly, both high-frequency and low-frequency conditions in Experiment 1 share the same data of beliefs about font size. Hence, to avoid data re-use, only the data from the high-frequency condition was included. Secondly, a set of experiments used low-frequency words as their main stimuli, but only a few used high-frequency words. Therefore, we decided to include high-frequency words’ data and exclude low-frequency words’ data of our Experiment 1 in the current meta-analysis (In S2 Appendix, the data of Experiment 1 was not included and the same meta-analysis on the remaining five studies was conducted. The results were very similar.).

The correlation between difference in beliefs between large and small words and difference in JOLs between the two types of words was conducted in each experiment. The correlation coefficient serves as the effect size. Because the variance depends strongly on the correlation, the current meta-analysts did not perform syntheses on the coefficients itself. Rather, the Fisher’s *r*-to-*z* transformed correlation coefficient was used [38]. All analyses were conducted via the R *metafor* package [39]. Specifically, we used the *rma* function to fit a random-effects model. The forest plot is presented in Fig 5.

The meta-analysis revealed that the correlation between beliefs about font size and the font size effect on JOLs was significantly greater than 0, Fisher’s *z* = 0.29 (*SE* = 0.08), *Z* = 3.49, *p* < .001, 95% CI = [0.13, 0.46]. Fisher’s *z* transformed to *r* is 0.28, 95% CI = [0.13, 0.43]. Heterogeneity between studies was low, *Q*(5) = 6.71, *p* = .24, *I*^*2*^ = 24.19%. Moderator (subgroup) analyses revealed that WF moderates the effect, *Q*(1) = 5.49, *p* = .02 (see Table 4). The correlation between beliefs about font size and the font size effect on JOLs was significant for low-frequency words, but was non-significant for high-frequency words. Further discussions are provided in General Discussion.

**Table 4:** Moderator (subgroup) analysis results.

Moderator	*k*	Fisher’s *z*	*SE*	*Z*	*p*	95% CI	*Q*_*B*_
WF							5.49*
High-frequency	2	0.06	0.12	0.53	.60	[-0.17, 0.30]	
Low-frequency	4	0.42	0.09	4.57	< .001	[0.24, 0.59]	

**Note.***k* = number of studies; *SE* = standard error; CI = confidence interval; *Q*_*B*_ = heterogeneity for between-levels moderator tests; WF = word frequency

* represents *p* < .05.

## General discussion

Previous studies demonstrated that participants’ pre-existing beliefs about font size contribute importantly to the font size effect on JOLs when font size is the only manipulated factor [4,6,11]. The current study goes beyond this to ask whether beliefs about font size contribute to the font size effect on JOLs in circumstances of multiple cues. In Experiment 1, a 2 (font size: large *vs*. small) * 2 (WF: high *vs*. low) within-subjects design was adopted, with WF manipulated within-subjects to serve as an additional cue to inform JOLs. The results showed that beliefs about font size play little role in the font size effect on JOLs in circumstances of multiple cues (font size & WF). In Experiment 2, WF was changed as a between-subjects variable, which no longer served as an apparent cue to inform JOLs. With font size as the only within-subjects manipulated factor, the results showed that beliefs about font size mediate the font size effect on JOLs in a pure list of low-frequency words, consistent with previous findings [4,6,11]. However, beliefs about font size did not mediate the font size effect on JOLs in a pure list of high-frequency words. Lastly, a small-scale meta-analysis provided further evidence that WF per se does moderate the contribution of beliefs about font size to the font size effect on JOLs.

### The font size effect on JOLs

In the current study, higher JOLs were given to large words than to small ones across two experiments, replicating the classic font size effect on JOLs [7]. The font size effect on JOLs did not differ significantly between Experiment 1, Experiment 2’s high-frequency group and Experiment 2’s low-frequency group, *F* (2,134) = 1.34, *p* = .27. However, there is a trend in magnitude that the font size effect was smaller in both Experiment 1 (*M* = 5.54, *SD* = 8.85) and Experiment 2’s high-frequency group (*M* = 5.48, *SD* = 8.21) than that in Experiment 2’s low-frequency group (*M* = 7.93, *SD* = 8.35). The individual-level analysis of cue utilization showed that 53.13%, 56.60%, 65.38% of participants in each of Experiment 1, Experiment 2’s high-frequency group, and Experiment 2’s low-frequency group reliably based JOLs on font size, as their JOLs were higher for large words than for small ones and Cohen’s *d* for the font size effect on JOLs was greater than 0.20 [15]. However, the proportions of participants did not differ significantly across experiments,*χ*^2^(2) = 1.46, *p* = .48.

### Contribution of beliefs about font size to the font size effect

Across two experiments in the current study, in the first-day belief questionnaire, higher predictions were given to large words than to small ones, reflecting that participants indeed held beliefs about how font size affects memory before they took the learning task. The key finding of the current study was that, although belief-based predictions and JOLs varied in the same direction across the two experiments, this does not necessitate to mean that beliefs about font size contribute to the font size effect on JOLs. Through the multilevel mediation analyses, the current study was the first one (as far as we know) to provide direct evidence that people’s pre-existing beliefs about font size suffer from limitations to account for the font size effect on JOLs in circumstance of multiple cues (Experiment 1) or in a pure list of high-frequency words (Experiment 2’s high-frequency group).

As only in a pure list of low-frequency words in Experiment 2 did we observe that beliefs about font size contribute to the font size effect on JOLs, it is critical to determine whether this finding is replicable. Indeed, an unpublished experiment conducted in our laboratory as well observed that beliefs about font size significantly mediate the font size effect on JOLs in a pure list of low-frequency words (See S1 Appendix for details).

### Explanation of results in conjunction with existing theories

While previous research has largely focused on how a single cue influences metamemory judgements, a recent study by Undorf and colleagues found that multiple cues can also jointly affect JOLs [15]. In their Experiment 1, font size and number of study presentations were simultaneously manipulated within-subjects. The results verified the font size effect on JOLs in circumstance of multiple cues. The current study goes further to ask whether the font size effect on JOLs in circumstance of multiple cues is driven by one’s pre-existing beliefs about font size. However, the documented results showed that this is not the case.

Why do beliefs about font size fail to act on the font size effect in circumstance of multiple cues? According to Mueller and colleagues’ speculation of the analytic-processing theory [14] (p. 13): “*If they (participants) can develop a plausible explanation for why a cue may influence memory or retrieve a previously developed explanation from long-term memory*, *then they will use belief about the cue as they make JOLs*. *If not*, *then other factors will affect JOLs—most notably the differences in processing fluency that may arise as people study each item*”. As the results from the first day’s belief questionnaire showed that participants indeed hold pre-existing beliefs that large words are easier to remember than small ones, we inferred that the absence of the belief effect is probably due to participants’ disregarding of beliefs about font size when making JOLs.

When participants are explicitly instructed to make JOLs, their goal is to search for any cues—or variability across items—that are plausibly related to memory. Participants may have multiple cues to base their JOLs on (e.g., superficial cues such as font size, semantic cues such as WF). When dealing with multiple cues in making JOLs, people might give different weight to different cues [15]. In Experiment 1, both font size and WF were manipulated as factors. Font size is a perceptual cue which is unrelated to recall performance [7], but WF is instead a diagnostic cue for predicting recall performance. Almost all participants (*N* = 30, 93.75%) used WF as a cue when making JOLs (Cohen’s *d* ≥ 0.20 for the WF effect on JOLs). In addition, half participants based JOLs on both font size and WF. Experiment 1 manipulated WF as another factor, which indeed attracted participants’ attention on WF. A tentative explanation is that participants might assign higher weight to WF than to font size, perhaps because WF is considered more diagnostic of future recall performance than font size. Participants might then pay less attention to font size and consequently might not take pains to retrieve a previously developed explanation about how font size influences memory performance. In this way, beliefs about font size could hardly account for the font size effect on JOLs.

One may ask what underlies the font size effect on JOLs in Experiment 1. A previous study has shown that large items are perceived more fluently than small ones [12]. In the current experiment, although font size did not influence JOLs in an analytic and conscious way (i.e., pre-existing beliefs that large words are easier to remember), it could influence JOLs through an implicit and unconscious way (i.e., large words are perceived more fluently), which could still produce the font size effect on JOLs. This speculation is quite similar with what Undorf and colleagues discussed [15]: “*It remains to be seen how analytic processing theory may fare in experiments with multiple varying cues*, *in which relevant beliefs are probably harder to activate or develop*. *Maybe*, *the availability of multiple cues fosters the reliance of JOLs on nonanalytic*, *experience-based processes such as fluency*”. The current study should be the first to demonstrate that beliefs about font size would contribute minimally to the font size effect on JOLs when WF was manipulated as another factor. Further studies are strongly recommended to test how participants assign different weight to different metamemory cues, and how could this change the mechanism underlying JOLs.

Another intriguing finding was that, in Experiment 2, beliefs about font size significantly mediated the font size effect in a pure list of low-frequency words, but not in a pure list of high-frequency words. The meta-analysis also revealed that the correlation between beliefs about font size and the font size effect on JOLs was moderated by WF. In Experiment 2, WF was changed into a between-subjects variable, which was controlled in a relatively small range in each of the high/low-frequency list. According to the analytic-processing theory, when font size serves as the only apparent cue for JOLs, participants are supposed to easily detect font size as an available cue and retrieve their *a priori* beliefs about how font size affects memory performance, which in turn drives the font size effect on JOLs. However, the results of Experiment 2 together with the meta-analysis showed that this is true for a pure list of low-frequency words, but not for a pure list of high-frequency words.

Previous studies have shown that high-frequency items have semantic advantages in being easier to process, to associate with one another, to bind to episodic contexts and to hold in working memory [21,23]. Compared to low-frequency words (e.g., Lime), high-frequency words (e.g., Hospital) have its own semantic characteristics. Firstly, high-frequency words are experienced more often in daily life, thus have strong existing traces in memory, and are easy to form and store episodic memory traces [40]. In addition, high-frequency words are more likely to have pre-existing semantic associations to one another, which can facilitate the formation of associations among high-frequency words in the study list [41]. As participants learned the high-frequency words in a pure list, they may find the words could be easily associated with their pre-existing experience (e.g., Hospital: I went to the Hospital yesterday). Moreover, they could form association among the words in the pure list of high-frequency words (e.g., Actor: I have just learned a word “Model”, both “Actor” and “Model” are occupations, so I can remember them together). However, such encoding process might be quite difficult when encountering a pure list of low-frequency words. Although the low-frequency words in our experiment are not novel words, they are not experienced frequently in daily life. In this way, participants might have difficulty to bind the just-learned word with their own previous experience or form associations among low-frequency words in the study list [40,41]. One possibility was that the semantic characteristics of words might also serve as cues to inform JOLs (semantic cues). For example, if one word could be associated with one’s own experience or could be associated with other words in the study list, it might receive higher JOLs. Although such semantic cues could not help generate different JOLs for different words in a pure list of high-frequency or low-frequency words, as all high-frequency words have strong semantic characteristics while all low-frequency words have weak semantic characteristics, it may influence how participants weight other cues (such as font size). One tentative explanation of Experiment 2 is that participants might give low weight to font size when making JOLs for a pure list of high-frequency words, just because they were attracted by the strong semantic cues associated with high-frequency words. With lower weight on font size when making JOLs, participants might not fully retrieve and utilize their pre-existing beliefs about font size, but be unconsciously influenced by the fluency difference induced by the font size manipulation to make different JOLs. However, in a pure list of low-frequency words, the semantic cues were weak. Participants might give higher weight to font size when making JOLs. In this way, participants might retrieve their pre-existing explanation of how font size influences memory to form JOLs.

The current study found indirect evidence for the possible difference of weight assigned to font size when making JOLs for high-frequency or low-frequency words. After deleting the influential observations in Experiment 2, the similar 2 (font size: large *vs*. small, within-subjects variable) * 2 (WF: high *vs*. low, between-subjects variable) mixed ANOVA was conducted, with JOLs as the dependent variable. The results showed that there was a significant interaction between font size and WF, *F* (1, 95) = 7.41, *p* = .008, *η*_p_^2^ = 0.07. The font size effect on JOLs was larger in low-frequency group than that in high-frequency group, indicating that participants might give higher weight to font size when making JOLs for a pure list of low-frequency words compared with a pure list of high-frequency words. However, the current study did not ask participants about what factor they base their JOLs on. Future studies are strongly encouraged to test this possibility through directly asking participants to report the cues they use to inform JOLs, and to investigate how participants assign different weights to different metamemory cues.

Another possible explanation is that the variability of WF for the study materials is high in both Experiment 1 (*SD* = 96.41) and Experiment 2’s high-frequency group (*SD* = 37.43), but is relatively low in Experiment 2’s low-frequency group (*SD* = 0.22). Therefore, it is possible that when there is small variability in WF, people seem to use belief-based process to make JOLs. If there is large variability in WF (as in Experiment 1 and Experiment 2’s high-frequency group), people do not seem to use belief-based process to make JOLs. One may suspect that, not the WF per se, but the difference in WF variability is responsible for different results documented in the current study. Limited by the Chinese word database, the current study could not find sufficient high-frequency words with extreme low variability in WF. To test this potential explanation, one method is to include WF variability as a predictor in the analysis, which could control its potential influence.

The *coefficient of variation* (*SD*/mean, relative *SD*) is a statistical measure of the dispersion of data points around the mean, which is a more reasonable measure of data variation especially when the means corresponding to different *SD*s differ substantially. In this way, *coefficient of variation* for WF was calculated for each of the six studies in the above meta-analysis, in order to represent WF variability in each study. Later, a similar meta-analysis was performed, with both WF (high *vs*. low) and WF variability as moderators. The results showed that WF variability did not significantly moderate the effect, *Q*(1) = 0.22, *p* = .64. After controlling the potential influence of WF variability, WF (high *vs*. low) still significantly moderated the correlation between beliefs about font size and the font size effect on JOLs, *Q*(1) = 5.51, *p* = .02. Therefore, WF variability might not fully explain the findings in our study, and it is reasonable to assume that WF per se might influence how beliefs about font size contribute to the font size effect, as mentioned above. Future studies should consider controlling for WF variability carefully to avoid potential influences on research findings.

### Limitations and future research direction

The current study suffers from several limitations. First, Experiment 1 did not include a control condition in which font size is the only manipulated factor and WF is controlled in a relatively small range. The current study compared the findings with both Hu et al. [4] and Su et al. [11] to make inferences that the manipulation of WF may be responsible for the absence of the contribution of beliefs about font size to the font size effect in Experiment 1. We acknowledged that it is better to include a control condition and replicate the belief-based font size effect, prior to interpret the absence of such belief effect when WF was manipulated as another factor.

Second, participants might have multiple beliefs or beliefs about the interaction effects among cues in circumstance of multiple cues. However, as the current study mainly focused on the relationship between beliefs about font size and the font size effect, we did not measure beliefs about WF and beliefs about the interaction between font size and WF. As a related issue, when participants encountered a high-frequency word presented in a small font size, their beliefs regarding font size and WF contradicted each other. That is, participants may have a pre-existing belief that high-frequency words would be remembered better. While they may also believe that words in small font size are difficult to remember. These two beliefs competed with each other. How did participants deal with multiple beliefs to form a JOL? In circumstance of multiple cues, participants may assign different weights to different beliefs in order to give a judgment. This question needs to be further investigated.

Third, the current study is the first to show the phenomenon that beliefs about a certain cue no longer contribute to the cue’s effect on JOLs in circumstance of multiple cues. More evidence regarding this phenomenon is required. It should be acknowledged that the current study suffers from limitations in providing direct evidence about why such beliefs effect is absent in certain conditions. However, we do provide several potential explanations, and future studies are encouraged to concentrate on the mechanisms underlying JOLs in circumstance of multiple cues.

Lastly, the current study is limited to answer whether people generally rely on *a priori* beliefs about the overall memory ability, or rely on the processing experience to make JOLs in circumstance of multiple cues, which awaits further exploration. Recently, Hu et al. [42] proposed a Bayesian inference model, which is the first computational framework to systematically account for how people integrate their current processing experience and *a priori* beliefs about their overall memory ability to evaluate memory performance. If people’s confidence about performance mainly relies on *a priori* beliefs about their overall memory ability, then their confidence ratings reported in memory tasks should be closely distributed around their *a priori* beliefs about memory ability, and variance of reported confidence ratings should be low. However, if processing experience plays an important role in the metamemory process, then variance of reported confidence ratings should be high. In other words, the Bayesian inference model could estimate the absolute contribution of processing experience and beliefs to metacognitive judgments. Future studies are encouraged to employ the Bayesian inference model to explore the mechanisms underlying JOL formation in circumstance of multiple cues.

In summary, even though previous studies have established that beliefs about font size contribute to the font size effect on JOLs when font size is the only within-subjects manipulated factor, the current study provides new evidence that the belief-based account suffers from limitations to account for the font size effect in certain circumstances. Further exploration of the belief-based account of JOL formation is valuable and will promote theory development for JOL construction.

## Supporting information

S1 Table Results of multilevel mediation model predicting JOLs of Hu et al.’s Experiment 2 and Su et al.’s Experiment 2b. (DOCX)

S1 Appendix The replicated experiment of Experiment 2’s low-frequency group. (DOCX)

S2 Appendix Meta-analysis that not include the data of Experiment 1. (DOCX)
