# Supplementary material for: The contribution of metamemory beliefs to the font size effect on judgments of learning: Is word frequency a moderating factor?
Source: PLoS One. 2021 Sep 20;16(9):e0257547. doi: 10.1371/journal.pone.0257547 (PMC8452059; doi:10.1371/journal.pone.0257547)
Supplement: S1 Table — (DOCX) [file pone.0257547.s001.docx]

**S1 Table.** **Results of multilevel mediation model predicting JOLs of Hu et al.’s Experiment 2 and Su et al.’s Experiment 2b**

| Effect | Estimate (*β*) | *SE* | *df* | *t* or *Z* value | *p* | 95% CI |
| --- | --- | --- | --- | --- | --- | --- |
| **Hu et al.’s Experiment 2** | | | | | | |
| *a* | 7.60 | 0.19 | 998 | 40.73 | < .001 | [7.23, 7.97] |
| *b* | 0.33 | 0.12 | 29.88 | 2.81 | 0.009 | [0.09, 0.57] |
| *c'* | 1.56 | 1.12 | 288.70 | 1.39 | 0.165 | [-0.64, 3.76] |
| *INDbelief* | 2.52 | 0.90 |  | 2.80 | 0.005 | [0.72, 4.28] |
| **Su et al.’s Experiment 2b** | | | | | | |
| *a* | 13.53 | 0.27 | 1073 | 50.22 | < .001 | [13.00, 14.06] |
| *b* | 0.77 | 0.27 | 27.86 | 2.89 | 0.008 | [0.22, 1.31] |
| *c'* | 2.73 | 4.29 | 27.92 | 0.64 | 0.53 | [-6.06, 11.51] |
| *INDbelief* | 10.37 | 3.60 |  | 2.88 | 0.004 | [3.30, 17.54] |

**Note.** *SE*, standard error; *df*, degree of freedom; CI, confidence interval; *INDbelief*, the indirect effect of *Font Size* on *JOL* through *Belief*. For *INDbelief*, *Z* value was reported.
