# Supplementary material for: The contribution of metamemory beliefs to the font size effect on judgments of learning: Is word frequency a moderating factor?
Source: PLoS One. 2021 Sep 20;16(9):e0257547. doi: 10.1371/journal.pone.0257547 (PMC8452059; doi:10.1371/journal.pone.0257547)
Supplement: S1 Appendix — (DOCX) [file pone.0257547.s002.docx]

**S1 Appendix. The replicated experiment of Experiment 2’s low-frequency group**

In this unpublished experiment (Experiment S1), the same 42 low-frequency words were used as those in the low-frequency group in Experiment 2. The only difference was that, before the study-test task, participants performed a lexical decision task. Following the completion of the lexical decision task, participants took the study-test task, which was the same as that in the low-frequency group in Experiment 2.

Participants in this study were 32 students recruited from BNU, with 22 females and mean age = 22.09 (*SD* = 2.31). Each participant was tested individually, received 35 RMB as compensation, and provided written consent.

The *Means* (*SD*s) of beliefs, JOLs, and recall performance for large and small words are presented in Table A. In the belief survey, participants estimated that they would recall more large than small words, *t*(31) = 5.54, *p* < .001, Cohen’s *d* = 0.98. In the study-test task, participants also made higher JOLs for large words than for small words, *t*(31) = 5.04, *p* < .001, Cohen’s *d* = 0.89. Recall performance did not differ significantly between large and small words, *t*(31) = 0.17, *p* = .86, Cohen’s *d* = 0.03.

**Table A. *Means* (*SDs*) of beliefs, JOLs, and recall performance in Experiment S1.**

|  | font size | |
| --- | --- | --- |
|  | large | small |
| Beliefs (%) | 48.75 (14.87) | 37.34 (13.14) |
| JOL (%) | 54.07 (15.59) | 47.11 (17.23) |
| Recall (%) | 45.00 (21.59) | 44.53 (21.49) |

As in Experiment 1, the R *olsrr* package was used to depict DFFITS to detect influential observations. In Experiment S1, four participants were detected as outliers and were excluded from a further regression analysis. For the remaining 28 participants, Fig A shows the relationship between the font size effects on beliefs and JOLs.


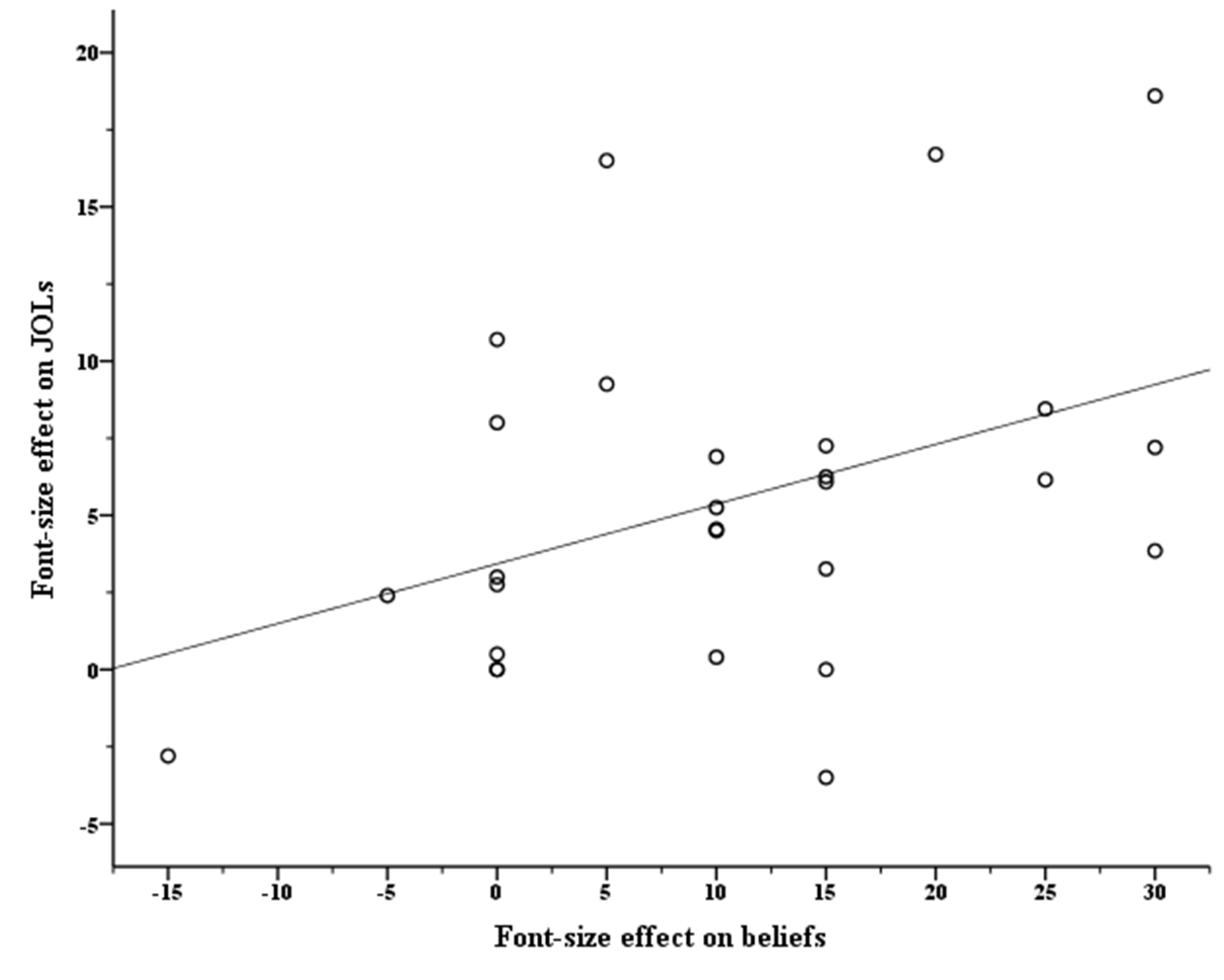


**Fig A. The relationship between the font size effects on beliefs and JOLs in Experiment S1.**

A similar multilevel mediation analysis as that in the low-frequency group in Experiment 2 was conducted. The coefficients were shown in Table B. The indirect effect of font size on JOLs through beliefs about font size was significant.

The data of Experiments S1 (*N* = 28, after excluding the 4 outliers) and those of the low-frequency group in Experiment 2 (*N* = 50, after excluding the 2 outliers) was combined. The variable *Group* was added as a participant-level moderator, where group = 0 represented the low-frequency group in Experiment 2, and group = 1 represented Experiment S1. A moderated mediation analysis, similar to that in Experiment 2, was conducted. The coefficients were shown in Table B. The indirect effect of font size on JOLs through beliefs about font size was significant. In addition, the same Bayesian linear regression was conducted as Experiment 1, in which the data of Experiments S1 (*N* = 28) and those of the low-frequency group in Experiment 2 (*N* = 50) was included. The data are 14.87 times more likely under a regression model including *difference in beliefs* compared to the null model (BF_10_ = 14.87). Another multilevel mediation analysis for all low-frequency words was conducted, in which we did not exclude any outliers from data analysis (*N* = 84). The results also showed that the indirect effect of font size on JOLs through beliefs about font size was significant. In this way, these consistent findings jointly support the claim that beliefs about font size contribute to the font size effect on JOLs in a pure list of low-frequency words.

**Table B. Results of multilevel mediation model predicting JOLs of Experiment S1, Experiment 2_low-frequency group & Experiment S1**

| Effect | Estimate (*β*) | *SE* | *df* | *t* or *Z* value | *p*-value | 95% CI |
| --- | --- | --- | --- | --- | --- | --- |
| **Experiment S1 (*N* = 28)** | | | | | | |
| a | 10.34 | 0.34 | 1114 | 30.83 | < .001 | [9.68, 11.00] |
| b | 0.19 | 0.10 | 1086.00 | 1.97 | 0.049 | [0.001, 0.39] |
| c' | 3.44 | 1.50 | 1086.00 | 2.29 | 0.02 | [0.50, 6.37] |
| *INDbelief* | 2.01 | 1.02 |  | 1.97 | 0.049 | [0.02, 4.04] |
| **Experiment 2_low-frequency group & Experiment S1 (*N*_low-frequency_ = 50, *N*_S1_ = 28 )** | | | | | | |
| a | 14.03 | 0.22 | 3102.00 | 65.01 | < .001 | [13.60, 14.45] |
| b | 0.22 | 0.07 | 76.88 | 3.14 | 0.002 | [0.08, 0.35] |
| c' | 4.16 | 1.21 | 76.55 | 3.45 | < .001 | [1.76, 6.57] |
| *Group* | 2.06 | 3.80 | 76.01 | 0.54 | 0.59 | [-5.51, 9.62] |
| *Group***Belief* | -0.05 | 0.10 | 75.13 | -0.55 | 0.59 | [-0.25, 0.14] |
| *INDbelief* | 3.03 | 0.96 |  | 3.14 | 0.002 | [1.19, 4.92] |

**Note.** *SE*, standard error; *df*, degree of freedom; CI, confidence interval; *INDbelief*, the indirect effect of *Font Size* on *JOL* through *Belief*. For *INDbelief*, *Z* value was reported.
