# Supplementary material for: The contribution of metamemory beliefs to the font size effect on judgments of learning: Is word frequency a moderating factor?
Source: PLoS One. 2021 Sep 20;16(9):e0257547. doi: 10.1371/journal.pone.0257547 (PMC8452059; doi:10.1371/journal.pone.0257547)
Supplement: S2 Appendix — (DOCX) [file pone.0257547.s003.docx]

**S2 Appendix. Meta-analysis that not include the data of Experiment 1**

We appreciate one anonymous reviewer’s opinion that Experiment 1 manipulated word frequency (WF) within-subjects, which is different from other studies, and might bias the results if including it in the meta-analysis. In this way, we excluded the data of our Experiment 1 and rerun the same meta-analysis on the remaining five studies as in the current study.

The meta-analysis revealed that the correlation between beliefs about font size and the font size effect on JOLs was significantly greater than 0, Fisher’s *z* = 0.34 (*SE* = 0.09), *Z* = 3.78, *p* < .001, 95% CI = [0.16, 0.51]. Fisher’s *z* transformed to *r* is 0.32, 95% CI = [0.16, 0.47]. Heterogeneity between studies was low, *Q*(4) = 4.78, *p* = .31, *I^2^* = 21.34%. Moderator (subgroup) analyses revealed that WF marginally moderates the effect, *Q*(1) = 3.60, *p* = .06 (see Table A), which is a little bit different from the results of including Experiment 1’s high-frequency condition. This marginal significant effect might result from the fact that only one effect of high-frequency condition was included (*k* = 1). However, we could still find that the correlation between beliefs about font size and the font size effect on JOLs was significant for low-frequency words, but was non-significant for high-frequency words. Overall, the results showed that whether or not including the data of Experiment 1 has minimal influence on the results.

**Table A. Moderator (subgroup) analysis results (after excluding the data of Experiment 1)**

| Moderator | *k* | Fisher's *z* | *SE* | *Z* | *p* | 95% CI | *Q_B_* |
| --- | --- | --- | --- | --- | --- | --- | --- |
| WF | | | | | | | 3.60^•^ |
| High-frequency | 1 | 0.08 | 0.15 | 0.54 | .59 | [-0.21, 0.38] |  |
| Low-frequency | 4 | 0.42 | 0.09 | 4.57 | < .001 | [0.24, 0.59] |  |

**Note.** *k* = number of studies; *SE* = standard error; CI = confidence interval; *Q_B_* = heterogeneity for between-levels moderator tests; WF = word frequency;

^•^ represents .05 < *p* < .10.
